# Body mass index moderates the relationship between C-reactive protein and depressive symptoms: evidence from the China Health and Retirement Longitudinal Study

**DOI:** 10.1038/srep39940

**Published:** 2017-01-27

**Authors:** Tingting Qin, Wenhua Liu, Minghui Yin, Chang Shu, Mingming Yan, Jianyuan Zhang, Ping Yin

**Affiliations:** 1 Department of Epidemiology and Biostatistics, School of Public Health, Tongji Medical College, Huazhong University of Science and Technology, Wuhan 430030, China; 2 Tongji Hospital, Tongji Medical College, Huazhong University of Science and Technology, Wuhan 430030, China

## Abstract

The present study aimed to investigate the role of abnormal body mass index (BMI, kg/m^2^) in the depression-CRP (C-reactive protein) relationship in a healthy middle-aged and elderly Chinese population. Analytical samples were drawn from the China Health and Retirement Longitudinal Study (CHARLS), and participants were categorized by different BMI levels. Depressive subtypes were evaluated both at baseline and follow-up using the Center for Epidemiology Studies Depression scale. Hs-CRP and other variables were measured at baseline. Multiple linear regression analyses were used to evaluate the cross-sectional and longitudinal relationship between depression and baseline hs-CRP. Depression was significantly negatively associated with BMI (*ρ* = −0.077, *p* < 0.0001), with underweight associated with worse depressive symptoms than other BMI groups. Both cross-sectional and longitudinal associations between serum hs-CRP and depressive subtypes were significantly positive in the underweight group (*p* < 0.05). However, in the other BMI groups (from normal weight to obesity), the CRP-depression relationship was no longer significant (*p* > 0.05). The significant relationship between CRP and depression in the underweight group suggested that not only obesity but also a low BMI could explain a substantial portion of the inflammation-depression link.

As the prevailing causes of disability and death have shifted from infectious to chronic non-communicable diseases, the disease burdens of middle-income countries, including China, can mainly be attributed to cardiovascular or neoplastic disease. Neuropsychiatric disorders, as the most important causes of these illnesses, have triggered a change in the thinking regarding international mental health and have attracted investigators’ attention^1^. “Currently, an estimated 10.5% of the total global burden of disease is attributable to neuropsychiatric conditions^2^. Furthermore, in China, the estimated prevalence of neuropsychiatric disorders is 17.5% (*95%CI* 16.6–18.5)^3^. In addition to this problematic prevalence, the burden of neuropsychiatric damage caused by immune disorders also has serious and unfavorable consequences. Mak *et al*. revealed that neuropsychiatric damage has adversely affected the survival of patients suffering from chronic immune disease from the 1950 s to the 2000s^4^. Furthermore, guidelines on the early recognition and management of different neuropsychiatric manifestations are still being established.” According to the World Health Organization, the most common mental disorder, i.e., depression, will be second only to heart disease by 2020 and will be the leading cause of disease burden by 2030^5^.

Depression is an independent risk factor for many chronic medical conditions^6^, including cardiovascular disease (CVD)^7^, diabetes^8^, high blood pressure^9^, asthma^10^, rheumatoid arthritis^11^, chronic obstructive pulmonary disease and systemic lupus erythematosus^12^. In particular, evidence of its longitudinal relationship with incident CVD, and especially its risk effects for CVD in the elderly, is abundant and consistent^7^,^13^,^14^. With respect to the complex pathophysiological mechanisms that could explain the role of depression in the development of CVD, as well as considering that depression can increase hemostatic factors and acute phase proteins such as C-reactive protein that lead to thrombus formation and myocardial infarction^15^, the chronic low grade systemic inflammation theory has been extensively documented. Furthermore, the inflammation-depression hypothesis has been assessed in many studies. Epidemiological and clinical studies have shown that systemic inflammatory markers are predictive of future incident cardiovascular events^16^. In addition, several meta-analyses have revealed that the relationship between increased inflammatory markers and depressive symptoms is significant^17^,^18^,^19^. However, as the adipose tissue of people with obesity secretes higher amounts of inflammatory molecules (e.g., CRP, TNF-α, IL-6, adiponectin and leptin) than the adipose tissue of lean individuals^20^, BMI has been reported to be a major mediator of the inflammation-depression relationship, leading to substantial variability in the conclusions across studies regarding the relationship between depression and inflammatory factors^21^,^22^,^23^. In a sample of 390 obese individuals with a BMI of 30–50 kg/m^2^, *ML Vetter* and colleagues found that major depression was significantly associated with serum hs-CRP in men^24^. However, given the unique study sample (clinical obesity and lacking underweight, normal weight and overweight people), their findings cannot determine whether this association would remain significant in other BMI categories. In another sample of 13,006 adults from the NHANES, Yuexing Liu *et al*. reported that adjusting for BMI was the major driving force behind the disappearance of the CRP-depression association. In addition, it has been suggested that abnormal body weight, both under- and overweight, explain a substantial part of the relationship between CRP and depression^25^. Furthermore, in a meta-analysis of cross-sectional studies, adjustment for BMI led to a substantial attenuation of the effect sizes for the association between CRP and depression. However, the change in longitudinal effects caused by adjusting for BMI is unknown. Moreover, considering the different cardiotoxicities of certain depressive subtypes^26^,^27^, some studies have also suggested that certain depressive subtypes may contribute to the variability in the inflammation-depression relationship. For instance, somatic symptoms were reported to be more strongly related to inflammatory markers than other depressive symptom clusters^28^,^29^.

To date, how BMI alters the etiological factors involved in the CRP-depression association remains unknown, and studies related to this topic across entire BMI ranges (from underweight to obese) are scarce. Therefore, further analyses are needed to identify the BMI-specific relationship between serum CRP and depression. The main objective of the present study was to evaluate the relationship between depression and serum hs-CRP across the entire BMI range (from underweight to obese) in a sample of middle-aged and elderly adults in the China Health and Retirement Longitudinal Study (CHARLS). In addition, we aimed to determine (1) whether the CRP-depression relationship was significant across the entire BMI spectrum in a non-Western population and (2) how the association between serum CRP and depressive symptom clusters varied when samples were classified as underweight, normal weight, overweight and obese.

## Results

### Descriptive statistics

The total number of participants in the final subsample for the cross-sectional analysis was 6091. Approximately 809 (13.84%) had elevated CRP levels, and 1908 (33.16%) suffered from major depressive symptoms at baseline. The sample characteristics in relation to different BMI levels at baseline are presented in Table 1. Obese, overweight, normal weight and underweight subjects differed by many factors. Men were more likely to be underweight, and women were more likely to be obese. In addition, BMI was negatively correlated with age (*ρ* = −0.210, *p* < 0.001), with underweight participants being older and obese participants being much younger. Moreover, subjects with obesity were more likely to have higher blood lipid levels, blood pressure (both diastolic and systolic) and abdominal adiposity, but they were less likely to suffer from chronic pain than subjects who were underweight.

**Figure 1:**
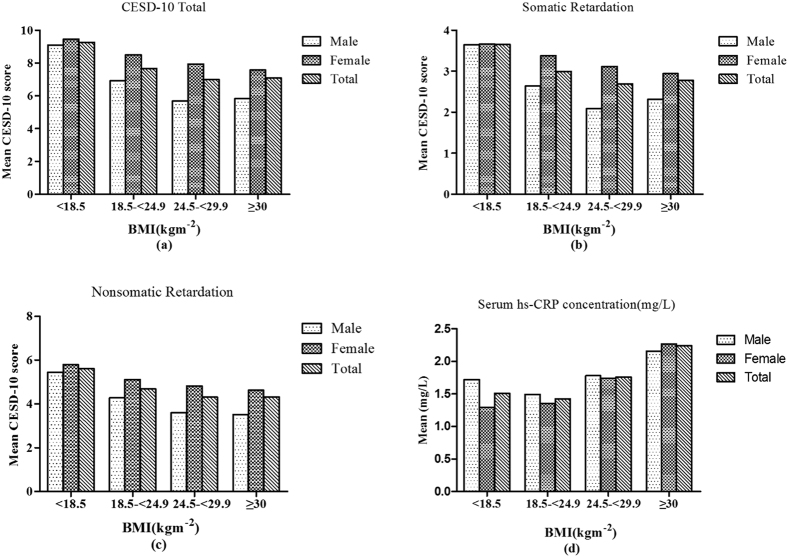
The mean CESD-10 scores of total (**a**), the mean CESD-10 scores of somatic retardation (**b**), the mean CESD-10 scores of non-somatic retardation (**c**), the mean serum hs-CRP concentrations (**d**) among Chinese adults aged ≥45 years by gender and BMI categories.

In addition, the prevalence of depression differed in the different BMI categories (χ^2^ = 31.857, *p* < 0.0001), and of the 1908 (33.16%) participants who suffered from clinically depressive symptoms (CESD-10 total score ≥10) at baseline, 43.88% were underweight, 33.73% were normal weight, 30.07% were overweight, and 27.27% were obese. Depression was negatively correlated with BMI (*ρ* = −0.077, *p* < 0.0001). In addition, the CESD-10 total score (*F* = 15.54, *p* < 0.0001), the somatic score (*F* = 13.69, *p* < 0.001) and the non-somatic score (*F* = 13.37, *p* < 0.001) were significantly higher in the underweight group than in the other BMI groups. Moreover, the gender difference in depression was not significant in underweight subjects, and the subscale depressive scores were approximately the same between underweight men and women (9.09 vs 9.46, *p* = 0.571, for CESD-10 total score). However, among the overweight and obese, women were more likely to be depressed than men (*p* < 0.01). The mean serum hs-CRP for the full sample and for each BMI group ranged from 1.4 to 2.3 mg/l, falling in the average cardiovascular disease risk category range of 1.0–3.0 mg/l^30^. The one-way ANOVA (*F* = 33.24, *p* < 0.001) also showed that serum hs-CRP differed between BMI groups, with the underweight having a lower serum hs-CRP mean concentration than the overweight (1.56 vs 1.86, *p* < 0.05) and the obese (1.56 vs 2.30, *p* < 0.05) but not differing from the mean concentration in the normal weight group (1.56 vs 1.44, *p* > 0.05). The results are shown in Fig. 1.

### Cross-sectional relationship of serum hs-CRP with depressive symptoms

A total of 6091 participants older than 45 years were included in the cross-sectional analysis. As shown in Table 2, analyses of the full sample showed that the association between CESD-10 total score and serum hs-CRP was not significant (*p* > 0.05 for all models). In addition, the somatic and non-somatic scores were not associated with serum hs-CRP in the full sample (*p* > 0.05 for all models). However, the analytical models stratified by BMI showed that CESD-10 total score was positively associated with serum hs-CRP among the underweight. Without adjusting for covariates, the coefficient of an SD increase in CESD-10 total score for each SD increase in log-transformed CRP at baseline was 0.109 (95%*CI* 0.033~0.184) in the underweight. However, in the other three BMI categories (from normal weight to obesity), the association was no longer significant (*p* > 0.05 for all). The same pattern of results was observed for somatic symptoms and non-somatic symptoms among the underweight. In addition, the somatic score was more strongly associated with serum hs-CRP (*β* 0.140, 95%*CI* 0.057~0.223) than the non-somatic (*β* 0.069, 95%*CI* −0.006~0.143) in the initial model. After controlling for covariates, the positive association between serum hs-CRP and somatic symptoms in the underweight group remained significant, with a coefficient and 95% confidence interval of 0.095 (0.088~0.102) for model 2 and 0.092 (0.085~0.099) for model 3. In addition, the association between serum hs-CRP and non-somatic symptoms was also significant, and the corresponding coefficient and 95% confidence interval was 0.032 (0.022~0.043) for model 2 and 0.018 (0.007~0.029) for model 3 among the underweight. However, no significant correlation between baseline depressive scores (including the CESD-10 total, somatic symptoms, and non-somatic symptoms) and serum hs-CRP was observed in the normal weight to the obese groups in any of the analytical models. In addition, results of the interaction test for gender showed no trend indicating a possible difference in the association between males and females (*estimates* = −0.0537, *std* = 0.0313, *p* = 0.0857).

### Longitudinal relationship of serum hs-CRP with depressive symptoms

Of the 4945 participants who remained in the longitudinal analysis, 617 did not have clinically depressive symptoms (CESD-10 total score < 10) at baseline but developed clinically depressive symptom two years later (CESD-10 total score ≥10), with 36 (22.22%) underweight, 384 (19.69%) normal weight, 165 (18.81%) overweight and 32 (22.07%) obese at baseline. The incidence of depression did not differ between different BMI categories (χ^2^ = 1.602, *p* = 0.659). As the modeling results for the longitudinal analyses show in Table 3, significant longitudinal associations between depression and baseline serum hs-CRP were also observed among the underweight after adjusting for covariates. The coefficient and 95% confidence interval for this relationship was 0.054 (0.028~0.079) for model 2 and 0.053 (0.023~0.084) for model 3. However, baseline serum hs-CRP was not associated with later depression in the normal weight to the obese groups (*p* > 0.05).

## Discussion

The aim of this study was to explore the cross-sectional and longitudinal associations between serum hs-CRP concentrations and depressive symptoms and to further test whether this association differed along the entire BMI spectrum (underweight, normal weight, overweight and obesity) in a large sample of the healthy middle-aged and elderly Chinese population. Total depressive symptoms, as well as somatic symptoms and non-somatic symptoms, were all strongly associated with serum hs-CRP in the underweight participants both cross-sectionally and longitudinally after adjusting for a range of potential confounders. Conversely, among the normal weight, overweight and obese participants, the pattern of results was identical to that of the full sample, which showed no significant relationship between depression and serum hs-CRP concentrations in any of the analytical models.

Interestingly, our study found that depressive symptoms were negatively associated with increased BMI levels. This was in contrast to most findings from Western studies, which have reported that clinical overweight or obesity is significantly associated with depression^31^,^32^,^33^,^34^. In addition, others have reported that both underweight and obesity are associated with depression, and a U-shaped relationship between depression and BMI has been described^35^,^36^. However, our findings are consistent with a previous study conducted in the Chinese elderly, which also demonstrated an inverse relationship between BMI and depressive symptoms. This relationship indicated that having a greater physiological and functional reserve from greater muscle mass in the elderly with a high BMI can protect against depressive symptoms^37^. Similarly, studies from other Asian populations have also reported that overweight or obesity was protective against depression. Shaheen Asghar *et al*. found that overweight individuals in Bangladesh had fewer symptoms of depression and concluded that obesity was an independent protective factor for depressive symptoms^38^. Another study from Hong Kong reported that obese elderly individuals were less likely to suffer from depressive symptoms than those of normal weight^39^. This counter-intuitive epidemiological result seems to be consistent with the “obesity paradox” theory, which reflects a relationship between obesity, compared with normal weight, and decreased mortality^40^. However, certain intrinsic biological connections, such as dysregulation of hormones, neurotransmitters and physiological functions, have been found in depression and obesity. Without considering these intrinsic biological links, nonbiological explanations may be more suitable for the opposite relationship observed in our study. It is possible that in the culture of most Western studies, obesity was stigmatized and represented being unhealthy. However, in some relatively less wealthy non-Western countries, such as China, a fuller body stature may be considered a sign of wealth and social status. In contrast, a thin body stature may reflect malnutrition, sub-health, or even poverty. Mood has been shown to be influenced by social, psychological, and cultural factors^41^. In addition, the CESD-10 mainly reflects one’s depressive experiences, which may be significantly influenced by mood. It is possible that different attitudes toward abnormal BMI, for instance, underweight and overweight, may affect one’s body satisfaction and self-esteem and thus affect one’s moods^42^.

The positive CRP-depression association that existed only in the underweight group in our study provided mixed evidence of the inflammation-depression research. Many previous studies have estimated the association between depression and serum CRP either cross-sectionally or longitudinally. However, studies related to the underweight or the entire BMI spectrum are scarce. Our research conducted the first attempt to explore how the CRP-depression association varied across subjects with underweight, normal weight, overweight and obesity and to clarify how abnormal body weight moderates the depression-inflammation relationship. Epidemiologic studies have demonstrated that not only obesity but also a low BMI can also be a risk factor for cardiovascular disease^43^, as a low BMI can increase the risk of impaired endothelium-dependent vasodilation through the increased oxidative stress^44^. In addition, when total depression was categorized as somatic symptoms and non-somatic symptoms, the depression-CRP relationship remained significant, suggesting that the inflammation-depression relationship cannot be changed by different depressive subtypes. Furthermore, of the 6091 subjects included in this study, none reported taking antidepressants or receiving psychotherapy. However, the reported prevalence of antidepressant use in the United States ranges from 46–60%^45^. In Europe, this prevalence ranges from 23–45%^46^, and in Australia, the prevalence ranges from 21–33%^47^. In addition to antidepressant use, psychotherapy such as cognitive behaviour therapy are also important for obese patients in western countries^48^. **A**ntidepressants could reduce inflammatory makers^49^ and psychotherapy could promote positive relationships, reduce maladaptive interpersonal behaviors and improve the symptoms of depression^50^. The use of antidepressant or psychotherapy could contaminate the results. But our study was free of these confounders and thus presented more reliable conclusions. General estimations of the use of antidepressants or psychotherapy in the Chinese population are scarce. However, the relatively low prevalence of antidepressant and psychotherapy use in this representative cohort indicated an existing difference in the treatment of depression between China and the West. These differences are worth further analyses.

There are several strengths of this study that should be noted. First, the CHARLS is a well-designed representative cohort study, and the large number of participants drawn from the general population enable the generalization of the conclusions to the middle-aged and elderly Chinese populations. Second, the study is the first to estimate the relationship between serum hs-CRP concentrations and depression across all BMI categories (from underweight to obesity) in a non-Western population. In addition, the findings have provided new evidence that confirms that abnormal body weight can moderate the inflammation-depression association. Third, the comprehensive set of sociodemographic, lifestyle, physical, metabolic and other factors related to depression provide adequate qualification to evaluate the association between serum CRP concentrations and depression. Consequently, these findings can supplement the findings of studies on the depression-inflammation relationship in the Asian population. Lastly, our participants are, on average, representative of the non-clinically depressed population and may be more generalizable and meaningful than some studies conducted in specific clinical populations. Thus, the findings of the present analysis may be applicable to the general healthy population.

It should also be noted that this studies has certain limitations. First, due to the missing data of CESD-10 scores and serum hs-CRP concentrations, large samples were excluded from the analysis. Additionally, there were relatively fewer samples with abnormal BMI levels (especially underweight and obese participants) compared with a normal BMI level in our study, and this type of unbalanced data may have introduced some bias. Therefore, applying new statistical methods, such as propensity score analyses, should be considered in subsequent analyses. Furthermore, our findings were based on both cross-sectional and longitudinal analyses, but blood samples were collected only once, at baseline. As a result, the longitudinal change in serum hs-CRP cannot be definitively established. In addition, the diagnoses of depressive symptoms were determined on the basis of the CESD-10 questionnaire rather than on the diagnoses of medical professionals. In addition, we used continuous CESD-10 scores for the analyses. Therefore, the present findings may not reflect clinically diagnosed episodes of depression. Finally, the present study focused only on the acute phase protein hs-CRP to explore the inflammatory-depression relationship. Future research should explore the role of other novel markers related to obesity and inflammation such as neuropeptide Y (NPY, a neurotransmitter responsible for increasing food intake and causing growth in fat tissue), adiponectin (a protein that regulates glucose levels and fatty acid breakdown) and pro-inflammatory cytokines (e.g., tumor necrosis factor-alpha (TNF-α) and interleukin-17). TNF-α and interleukin-17 have been reported to be involved in the pathophysiology of chronic diseases^51^. Lu *et al*.^52^ found that the mean BMI level was higher among patients with asthma than among healthy controls. In addition, in asthma patients with a high BMI, NPY level was positively correlated with adiponectin and TNF-α. These findings indicate that these meaningful markers are worth further investigation and should be addressed in future studies.

Notwithstanding its limitations, this study does provide evidence of cross-sectional and longitudinal relationships between depression and CRP in the underweight from a non-Western country, after adjusting for known confounders. Adiposity may play an etiological role in the connection between inflammatory activity and depression, but low BMI can also influence these associations. Therefore, it is worth focusing on the underweight to identify the metabolic pathways involved in the development of depression. In addition, further studies are needed to elucidate the role of low BMI level based on these findings.

## Methods

### Study Sample and Procedures

**Figure 2:**
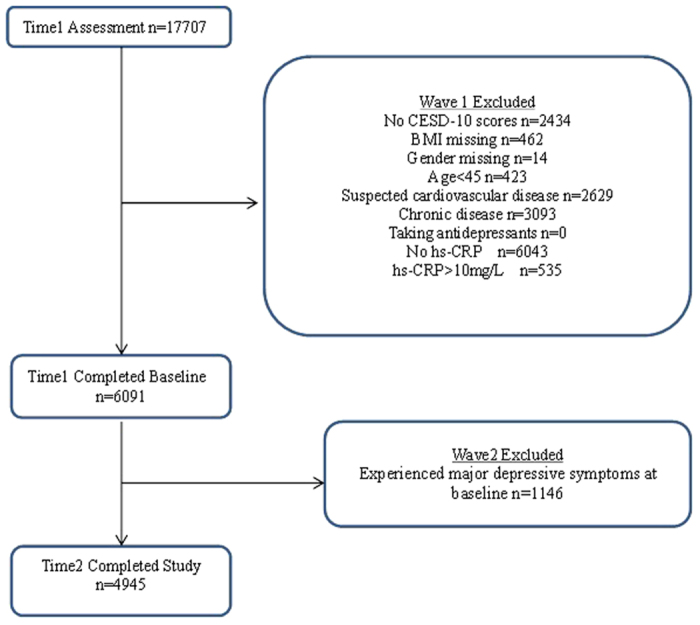
Flow chart of patients through the study.

The China Health and Retirement Longitudinal Study (CHARLS) is an ongoing nationally representative longitudinal cohort study of middle-aged and elderly adults in China that is managed by the National School for Development (China Center for Economic Research) at Peking University (PKU). Consistent with the U.S. Health and Retirement Study (HRS) family of surveys that study aging, the CHARLS is publicly available and de-identified. Respondents are derived from households with members aged 45 years or above, and the variables assessed can provide a wide range of information from socio-economic status to health conditions to better serve the scientific research needs of the middle-aged and elderly population. Samples are selected using multistage stratified probability sampling. The final sample contained 150 counties within 28 provinces. The respondents are followed every 2 years, and face-to-face computer-assisted personal interviews (CAPIs) are used to collect the information^53^. Detailed descriptions of the survey design and procedures are available at the study website (http://charls.ccer.edu.cn/zh-CN) or through the original study report^54^. The present analysis was based on the samples of the national baseline survey (visit I, 2011y) and the first follow-up survey (visit II, 2013y). Approximately 17,707 individuals participated in the first visit. We selected those aged 45 years and older and those with complete data on depression score, serum hs-CRP and BMI for inclusion in this analysis. As some specific conditions were likely to influence serum hs-CRP levels, we excluded 3093 participants who reported a history of cardiovascular disease (including myocardial infarction, coronary heart disease, angina, stroke, and other heart problems), chronic lung disease (including chronic bronchitis), liver disease, kidney disease, and arthritis or rheumatoid arthritis. As some participants may not have received a clinical diagnosis of heart problems, 2629 participants who reported pain on the left side of their chest or chest pains when climbing stairs, going uphill, or walking quickly were also excluded for suspected cardiovascular disease. Considering the fact that the use of antidepressants could reduce inflammatory makers and thus contaminate the results^49^, those receiving anti-depressants were also excluded. Additionally, 535 participants were excluded for having serum hs-CRP levels ≥ 10 mg/L, as values above this point are likely due to acute infection^30^. The final analytic sample that was entered into the cross-sectional analysis comprised 6091 individuals, with 2935 (48.18%) men and 3157 (51.82%) women. Excluding those without a follow-up depression score, 4945 participants remained in the longitudinal analysis. The biomedical ethics committee of Beijing University approved the study and informed consents was obtained from all participants. And all methods were performed in accordance with the relevant guidelines and regulations. A flow chart of patients through the study is presented in Fig. 2.

### Depressive Symptoms

Symptoms of depression were assessed with the Center for Epidemiological Studies Depression 10 Scale (CESD-10) scale^55^,^56^ both at baseline and at the follow-up visit. Participants were asked to rate their depressive symptoms and whether they had experienced any of the following in the past week: feeling bothered, having trouble concentrating, feeling depressed, feeling as though everything was effortful, feeling hopeful, feeling fearful, having restless sleep, feeling happy, feeling lonely, and having difficulty getting going. Each item was scored on a 4-point scale (ranging from “rarely or none of the time” = 0 to “all of the time” = 3), and the items were summed to produce a total score ranging from 0 to 30. Two positive items (item 5 and 8) were negatively worded and thus reversed-coded prior to analysis. The higher the score, the more serious the depression. The CESD-10 has been shown to be more appropriate for the nonclinical, general population^55^. According to prior studies that explored the factor structure of the CESD-10 in adolescents, we computed our depressive subscale scores based on the three-factor model proposed by Cheng and colleagues^57^, in which the items are separated into “positive affect” (PA, items 5 and 8), “depressive affect” (DA, items 1, 3, 6, and 9), and “somatic symptoms” (SR, items 2, 4, 7, and 10). In addition, the “positive affect” and “depressive affect” were recategorized as non-somatic symptoms. The CESD- 10 in this study has shown good internal consistency with α = 0.793 at baseline investigation (2011y) and α = 0.787 at the follow-up investigation (2013y).

### Blood and hs-CRP Data

Blood samples were collected (92% of respondents reported that they had fasted) during the investigation, and some blood-related indicators were measured. Venous blood was processed and divided into the plasma and buffy coat. Plasma samples were then transferred into three 0.5 mL cryogenic vials and immediately stored frozen at *−*20 °C before being transported to the Chinese CDC in Beijing within 2 weeks, where they were placed in a deep freezer and stored at −80 °C until being assayed at the Youanmen Center for Clinical Laboratory of Capital Medical University. C-reaction protein was measured from the frozen plasma using immunoturbidimetric assay. The detection limit was 0.1–20 mg/L, and the coefficient of variation was 5.7%^58^.

### Other factors

Age, gender, educational level, and marital status were obtained through a self-reported questionnaire at visit I. Education was assessed by asking the participants to report the highest level of educational qualification that they had attained. In addition, the twelve possible responses were categorized into four mutually exclusive groups: (1) those who never went to school and could neither read nor write were defined as “illiterate”; (2) those who had attended primary school or were reported to have been in “Sishu” were defined as “elementary school”; (3) those who had completed middle school were defined as “Middle school”; and (4) those who had completed high school, vocational school or college were defined as “High school and above”. Alcohol consumption was categorized as “current drinker” and “never drinker”. For current drinkers, those who drank liquor, beer or other alcoholic beverage more than once a week in the last year were classed as “regular drinkers”, and those consuming no more than once per month were classed as “occasional drinkers”. Smoking status was categorized as “current smoker”, “ex- smoker”, and “never smoker”. Marital status was defined as married, divorced, widowed and never married. Height, weight, waist circumference, pulse, and blood pressure were collected by professionally trained investigators using specific measurement tools. Systolic blood pressure, diastolic blood pressure and pulse were recorded three times, and the mean value of each subject was then calculated^59^. BMI was derived from height and weight based on the standard formula kg/m^2^. In addition, the sample was divided into underweight (BMI <18.5 kg/m^2^), normal weight (18.5 ≤ BMI < 25.0 kg/m^2^), overweight (25.0 ≤ BMI <30.0 kg/m^2^) and obese (30.0 kg/m^2^ ≤ BMI) according to the World Health Organization criteria. Information on some chronic diseases and chronic body pain was also gathered at the first visit. Hypertension was defined as having a mean systolic blood pressure ≥140 mmHg and/or a mean diastolic blood pressure ≥90 mmHg; taking antihypertension medication; or having any self-reported history of diagnosed hypertension^60^. Diabetes was defined as having a fasting plasma glucose level ≥126 mg/dl (7.0 mmol/L) and/or HbA1c ≥6.2 mmol/L^61^; using any treatment to control blood sugar regardless of fasting plasma glucose levels; or having any self-reported history of diagnosed diabetes^62^. Chronic body pain was assessed by asking the participants if they often suffered from pain and whether most of the time the pain was mild, moderate or severe. Only those with moderate or severe pain were classified as suffering from chronic pain.

### Statistical analysis

The study participants were stratified by BMI to describe the baseline characteristics. Continuous variables were presented as the means and standard deviations (SDs), and categorical variables were reported as the percentage (n (%)). The scores of the depressive symptom subscales and serum hs-CRP were natural log-transformed for normalization prior to statistical analysis. One-way analysis of variance (ANOVA) with *Bonferroni post hoc* test was performed to assess the differences in CES-D 10 scores and serum hs-CRP between the BMI groups.

Cross-sectional and longitudinal analyses of depressive symptoms and baseline hs-CRP concentrations were assessed using multiple linear regression analyses. All regression analyses were performed with CESD-10 scores as the criterion variable and serum hs-CRP as the predictor variable. In addition, the coefficients of an SD increase in CESD-10 score for each SD increase in serum hs-CRP at baseline were calculated. To avoid overfitting the models, only covariates that were associated with depression were included in the multivariable analysis. The initial model was used to analyze the raw associations between depression and serum hs-CRP without adjusting for any confounders. The second model consisted of the stepped entry of sociodemographic variables including age, sex, education, marital status, smoking status and alcohol consumption based on the initial model. The third model consisted of the stepped entry of additional health-related covariates including high-density lipoprotein, total cholesterol, and certain comorbidities such as diabetes, chronic pain and hypertension on top of the second model. The three model types specified above were used in the longitudinal analyses with CESD-10 scores at follow-up as the criterion variable. Considering the influence of baseline depression, estimations of the longitudinal association between later depression and baseline hs-CRP concentrations were conducted while controlling for baseline depression scores separately in the corresponding analytical models. Furthermore, to estimate whether specific depressive subtypes were more strongly associated with serum hs-CRP, we also separately included depressive subtypes, including somatic symptoms and non-somatic symptoms, in the cross-sectional and longitudinal regression analyses.

Given the reported gender difference^24^,^63^ of depression-CRP association, we also conducted exploratory analyses to test the interactions between serum hs-CRP and gender. All statistical analyses were weighted using the CHARLS sample weights, which considered the complex survey design and the differential response rates, and were performed with SAS (version 9.2.1, SAS Institute, Inc., Cary, NC). Two-sided hypothesis testing was used for all analyses, and a predetermined level of *p* < 0.05 was considered statistically significant.

## Additional Information

**How to cite this article**: Qin, T. *et al*. Body mass index moderates the relationship between C-reactive protein and depressive symptoms: evidence from the China Health and Retirement Longitudinal Study. *Sci. Rep.* **7**, 39940; doi: 10.1038/srep39940 (2017).

**Publisher's note:** Springer Nature remains neutral with regard to jurisdictional claims in published maps and institutional affiliations.

## Figures and Tables

**Table 1:** Baseline characteristics of the study sample stratified by BMI levels in the 2011 national survey (N = 6091).

Variable	Total Sample (6091)	Underweight (370)	Normal weight (3820)	Overweight (1640)	Obesity (261)
Age, years	58.82 (0.17)	65.09 (0.50)	59.14 (0.19)	57.09 (0.25)	56.5 (0.40)
Male, n (%)	2934 (47.72)	191 (50.30)	1993 (51.35)	679 (41.81)	71 (27.10)
Marital status					
Married, n (%)	5449 (88.37)	314 (81.31)	3381 (87.14)	1517 (92.70)	237 (89.69)
Divorced, n (%)	32 (0.52)	2 (0.43)	23 (0.65)	5 (0.23)	2 (0.66)
Widowed, n (%)	574 (10.39)	51 (17.32)	389 (11.31)	113 (6.77)	21 (9.26)
Never married, n (%)	36 (0.72)	3 (0.94)	27 (0.91)	5 (0.29)	1 (0.39)
Education					
Illiterate, n (%)	1650 (27.56)	144 (40.22)	1031 (27.56)	405 (24.70)	70 (27.01)
Finished elementary school, n (%)	2482 (40.20)	148 (39.75)	1625 (41.84)	612 (36.94)	97 (37.12)
Middle school, n (%)	1302 (21.67)	52 (13.77)	775 (21.02)	405 (24.30)	70 (26.44)
High school or above, n (%)	657 (10.57)	26 (6.26)	389 (9.58)	218 (14.06)	24 (9.43)
Smoking status					
Current smokers, n (%)	3678 (60.84)	195 (54.04)	2168 (57.46)	1113 (67.90)	202 (76.57)
Former smokers, n (%)	505 (8.16)	29 (7.26)	299 (7.69)	156 (0.79)	21 (8.39)
Never smokers, n (%)	1906 (31.01)	146 (38.71)	1351 (34.86)	371 (22.65)	38 (15.04)
Alcohol consumption					
Regular drinkers, n (%)	3692 (74.57)	230 (77.46)	2224 (72.60)	1052 (77.12)	186 (82.47)
Occasional drinkers, n (%)	352 (6.93)	18 (6.56)	222 (7.02)	102 (7.18)	10 (4.62)
Never drinkers, n (%)	965 (18.50)	52 (15.98)	660 (20.38)	224 (15.70)	29 (12.90)
Diabetes, n (%)	807 (13.09)	29 (6.98)	429 (11.44)	290 (16.97)	59 (22.12)
Hypertensive, n (%)	340 (5.58)	4 (1.95)	138 (3.64)	156 (9.24)	42 (16.77)
Suffering chronic pain, n (%)	1168 (18.73)	86 (22.99)	704 (18.04)	331 (19.24)	47 (19.29)
Abdominal Adiposity, n (%)	3053 (50.59)	17 (4.37)	1321 (35.56)	1469 (89.74)	246 (94.60)
Triglycerides (mg/dl)	135.08 (1.74)	99.4 (2.55)	122.6 (1.81)	166.15 (4.34)	169.81 (4.83)
Total Cholesterol (mg/dl)	193.35 (0.55)	186.81 (1.65)	191.38 (0.67)	198.36 (1.02)	199.44 (1.48)
Hdl Cholesterol (mg/dl)	50.62 (0.21)	59.14 (0.71)	52.55 (0.26)	45.28 (0.32)	44.5 (0.46)
Ldl Cholesterol (mg/dl)	116.29 (0.50)	109.57 (1.39)	115.27 (0.6)	119.39 (0.91)	121.04 (1.4)
Systolic (mmHg)	131.44 (0.39)	127.93 (1.12)	128.92 (0.4)	136.46 (0.73)	141.06 (1.73)
Diastolic (mmHg)	76.24 (0.18)	72.64 (0.5)	74.61 (0.2)	79.8 (0.31)	82.28 (0.71)
Pulse (bpm)	72.05 (0.15)	72.54 (0.47)	71.48 (0.17)	72.87 (0.27)	74.42 (0.53)
CESD −10 total score*	7.33 (0.07)	9.41 (0.26)a,b,^c^	7.40 (0.10)^b^	6.77 (0.13)	7.05 (0.27)
Somatic retardation Score*	2.88 (0.04)	3.73 (0.11)a,b,^c^	2.93 (0.05)^b^	2.61 (0.07)	2.67 (0.12)
Non-somatic retardation Score*	4.45 (0.05)	5.68 (0.18)a,b,^c^	4.47 (0.07)^b^	4.16 (0.09)	4.37 (0.17)
C-Reactive Protein (CRP) (mg/l)	1.60 (0.03)	1.56 (0.08)	1.44 (0.03)	1.86 (0.05)	2.30 (0.09)
CRP > 3	809 (13.84)	52 (15.29)	434 (11.62)	255 (16.57)	68 (27.53)
Baseline CESD −10 total score > 10	1908 (33.16)	154 (43.88)	1229 (33.73)	456 (30.07)	69 (27.27)

*Note.* Continuous variables are present as mean (standard deviation), and categorical variables are presented as percentage. Underweight is defined as BMI < 18.5 kg/m^2^; Normal weight is 18.5 ≤ BMI < 25 kg/m^2^; Overweight is 25 ≤ BMI < 30 kg/m^2^; Obesity is BMI ≥ 30 kg/m^2^.

^a^Significant different from normal weight group.

^b^Significant different from overweight group.

^c^Significant different from obesity group.

^*^Log-transformed by +1.

**Table 2:** Cross-sectional analysis of the relationship between continuous CESD - 10 scores and log-transformed CRP concentrations at baseline (2011y).

	Total (n = 6091)		Under weight (n = 370)		Normal weight (n = 3820)		Over weight (n = 1640)		Obesity (n = 261)	
	β(95%CI)	p-value	β(95%CI)	p-value	β(95%CI)	p-value	β(95%CI)	p-value	β(95%CI)	p-value
CESD-10 total										
Model 1	0.010 (−0.016, 0.036)	0.458	0.109 (0.033, 0.184)	0.005	0.022 (−0.012, 0.055)	0.203	0.004 (−0.052, 0.059)	0.895	−0.055 (−0.170, 0.060)	0.343
Model 2	−0.002 (−0.035, 0.030)	0.887	0.062 (0.052, 0.071)	<0.001	0.009 (−0.034, 0.053)	0.684	−0.053 (−0.116, 0.009)	0.096	−0.064 (−0.222, 0.095)	0.431
Model 3	−0.004 (−0.036, 0.028)	0.811	0.048 (0.038, 0.058)	<0.001	0.007 (−0.037, 0.051)	0.755	−0.058 (−0.119, 0.003)	0.064	−0.049 (−0.217, 0.120)	0.57
Somatic retardation										
Model 1	0.004 (−0.024, 0.032)	0.762	0.140 (0.057, 0.223)	<0.001	0.015 (−0.017, 0.046)	0.352	−0.016 (−0.083, 0.051)	0.639	−0.047 (−0.161, 0.066)	0.414
Model 2	−0.011 (−0.045, 0.023)	0.520	0.095 (0.088, 0.102)	<0.001	−0.006 (−0.047, 0.034)	0.755	−0.060 (−0.130, 0.010)	0.095	−0.001 (−0.146, 0.143)	0.984
Model 3	−0.016 (−0.049, 0.018)	0.358	0.092 (0.085, 0.099)	<0.001	−0.009 (−0.049, 0.031)	0.671	−0.076 (−0.142,−0.011)	0.022	0.003 (−0.143, 0.149)	0.971
Non-somatic retardation										
Model 1	0.014 (−0.011, 0.039)	0.272	0.069 (−0.006, 0.143)	0.071	0.026 (−0.007, 0.058)	0.118	0.010 (−0.040, 0.059)	0.701	−0.067 (−0.175, 0.041)	0.225
Model 2	0.009 (−0.023, 0.040)	0.596	0.032 (0.022, 0.043)	<0.001	0.024 (−0.018, 0.066)	0.257	−0.044 (−0.107, 0.019)	0.174	−0.089 (−0.232, 0.054)	0.222
Model 3	0.009 (−0.023, 0.041)	0.581	0.018 (0.007, 0.029)	0.001	0.023 (−0.019, 0.066)	0.287	−0.042 (−0.107, 0.023)	0.201	−0.083 (−0.231, 0.066)	0.275

Abbreviations: CESD, Chinese form of Center for Epidemiology Studies Depression scale; CRP, C-reactive protein; SE: standard error.

Underweight: BMI < 18.5 kg/m^2^; Normal weight: 18.5 ≤ BMI < 25 kg/m^2^; Overweight: 25 ≤ BMI < 30 kg/m^2^; Obesity: BMI ≥ 30 kg/m^2^.

Model 1: adjustment for no covariates.

Model 2: adjustment for age, sex, education, marital status, smoking status, alcohol consumption.

Model 3: adjustment for age, sex, education, marital status, smoking status, alcohol consumption, high density lipoprotein, total cholesterol, diabetes, hypertension, suffering chronic pain.

**Table 3:** Longitudinal analysis of the relationship between depressive symptoms at follow-up and log-transformed CRP concentrations at baseline (2011y).

	Total (n = 4945)		Under weight (n = 278)		Normal weight (n = 3092)		Over weight (n = 1362)		Obesity (n = 213)	
	β(95%CI)	p-value	β(95%CI)	p-value	β(95%CI)	p-value	β(95%CI)	p-value	β(95%CI)	p-value
CESD-10 total										
Model 1	0.014 (−0.014, 0.041)	0.326	0.071 (−0.013, 0.155)	0.097	0.028 (−0.005, 0.060)	0.100	0.010 (−0.053, 0.073)	0.765	0.056 (−0.079, 0.191)	0.414
Model 2	0.023 (−0.009, 0.056)	0.155	0.075 (0.048, 0.103)	<0.001	0.012 (−0.029, 0.053)	0.577	0.040 (−0.027, 0.106)	0.242	0.026 (−0.051, 0.102)	0.511
Model 3	0.023 (−0.009, 0.055)	0.156	0.066 (0.031, 0.102)	<0.001	0.016 (−0.025, 0.057)	0.437	0.033 (−0.032, 0.099)	0.317	0.032 (−0.054, 0.118)	0.467
Somatic retardation										
Model 1	0.012 (−0.019, 0.043)	0.451	0.039 (−0.065, 0.143)	0.461	0.016 (−0.019, 0.050)	0.376	0.026 (−0.050, 0.102)	0.507	0.060 (−0.094, 0.214)	0.444
Model 2	0.018 (−0.019, 0.055)	0.335	0.092 (0.072, 0.113)	<0.001	0.004 (−0.039, 0.048)	0.85	0.048 (−0.025, 0.122)	0.197	−0.024 (−0.094, 0.046)	0.496
Model 3	0.011 (−0.025, 0.048)	0.547	0.065 (0.037, 0.092)	<0.001	−0.000 (−0.043, 0.043)	0.999	0.043 (−0.030, 0.116)	0.249	−0.080 (−0.169, 0.009)	0.078
Nosomatic retardation										
Model 1	0.020 (−0.006, 0.045)	0.133	0.075 (−0.014, 0.165)	0.098	0.029 (−0.002, 0.060)	0.069	0.022 (−0.034, 0.079)	0.441	0.042 (−0.096, 0.180)	0.548
Model 2	0.030 (−0.001, 0.062)	0.055	0.054 (0.028, 0.079)	<0.001	0.016 (−0.023, 0.055)	0.424	0.044 (−0.020, 0.108)	0.178	0.077 (−0.000, 0.153)	0.051
Model 3	0.034 (0.003, 0.065)	0.032	0.053 (0.023, 0.084)	<0.001	0.024 (−0.015, 0.063)	0.221	0.040 (−0.024, 0.104)	0.224	0.112 (0.018, 0.206)	0.021

Abbreviations: CESD, Chinese form of Center for Epidemiology Studies Depression scale; CRP, C-reactive protein; SE: standard error.

Underweight: BMI < 18.5 kg/m^2^; Normal weight: 18.5 ≤ BMI < 25 kg/m^2^; Overweight: 25 ≤ BMI < 30 kg/m^2^; Obesity: BMI ≥ 30 kg/m^2^.

Model 1: adjustment for no covariates.

Model 2: adjustment for age, sex, education, marital status, smoking status, alcohol consumption, baseline CESD-10 score.

Model 3: adjustment for age, sex, education, marital status, smoking status, alcohol consumption, high density lipoprotein, total cholesterol, diabetes, hypertension, suffering chronic pain, baseline CESD-10 score.
